# Supplementary material for: Ginseng-Derived Carbon Quantum Dots Enhance Systemic Exposure of Bioactive Ginsenosides and Amplify Energy Metabolism in Mice
Source: Pharmaceutics. 2025 Nov 17;17(11):1485. doi: 10.3390/pharmaceutics17111485 (PMC12655082; doi:10.3390/pharmaceutics17111485)
Supplement: Supplementary file 1 [file pharmaceutics-17-01485-s001.zip › pharmaceutics-3952236-supplementary.pdf]

pharmaceutics

MDPI

Article

Ginseng-Derived Carbon Quantum Dots Enhance Systemic Exposure of Bioactive Ginsenosides and Amplify Energy Metabolism in Mice

Huiqiang Liu [1], Xin Sun [1], Bo Yang [1], Chuan Lin [1], Xiwu Zhang [1], Hui Sun [1,2], Xiangcai Meng [1,*], Yufeng Bai [3], Tao Zhang [3], Guangli Yan [1], Ying Han [1] and Xijun Wang [1,2,*]

1 State Key Laboratory of Integration and Innovation of Classical formula and Modern Chinese Medicines, National Chinmedomics Research Center, National TCM Key Laboratory of Serum Pharmacochemistry, Metabolomics Laboratory, Department of Pharmaceutical Analysis, Heilongjiang University of Chinese Medicine, Heping Road 24, Harbin 150040, China. 2660948835@qq.com (H.L.); 1219370452@qq.com (X.S.); yangbo@hljucm.edu.cn (B.Y.); 15983955737@163.com (C.L.); zhangxiwu@hljucm.edu.cn (X.Z.); sunhui@hljucm.edu.cn (H.S.); yanguangli@hljucm.edu.cn (G.Y.); hanying@hljucm.edu.cn (Y.H.)

2 State Key Laboratory of Quality Research in Chinese Medicine, Macau University of Science and Technology, Macau 999078, China.

3 Harbin Chengcheng Institute of Life and Material, Harbin 150040, China. baiyufeng@buaa.edu.cn (Y.B.); zhangtao@buaa.edu.cn (T.Z.)

* Correspondence: mengxiangcai@hljucm.edu.cn(X.C.M.); xijunw@sina.com(X.J.W.)

Supplementary material

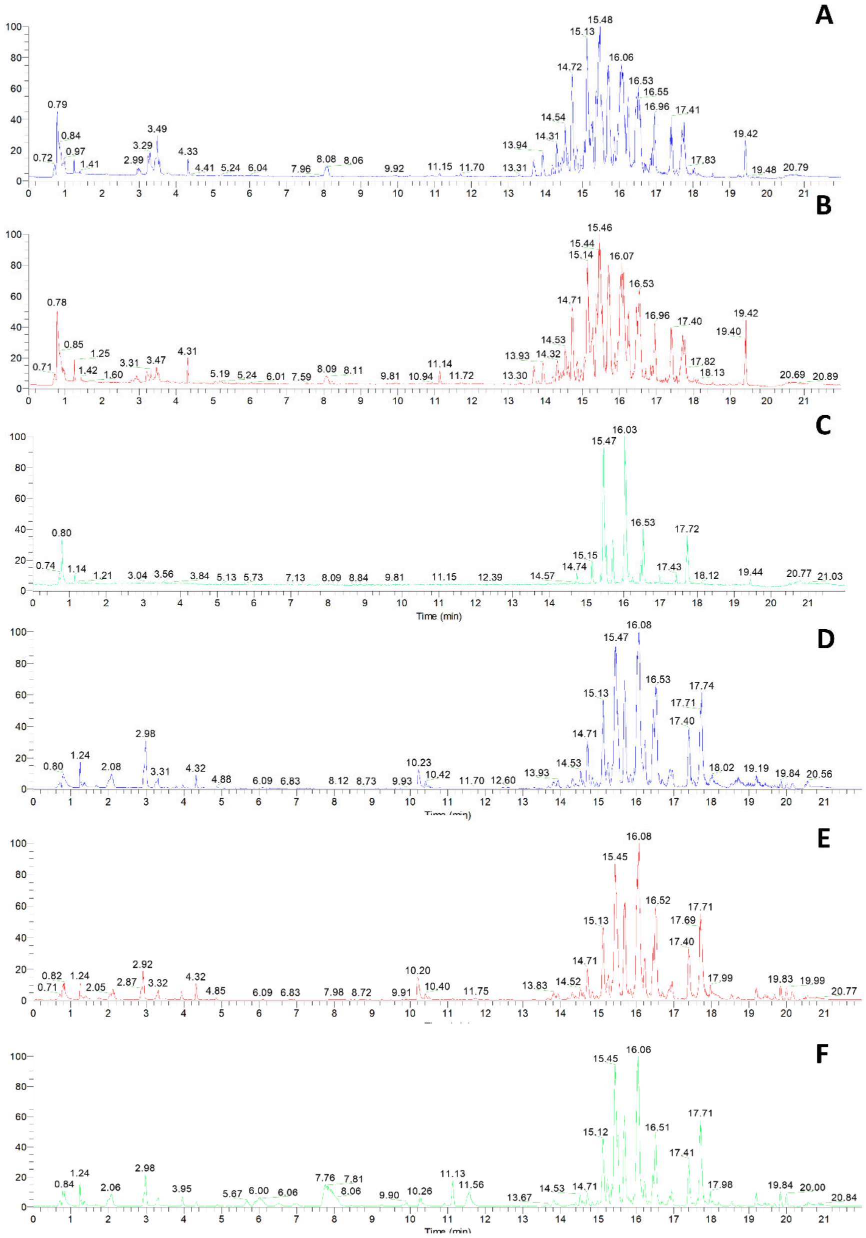

Figure S1. chromatograms of serum samples under positive/negative ionization modes (**A**) G-CQDs group (positive mode); (**B**) G-AE group (positive mode); (**C**) Control group (positive mode);(**D**) G-CQDs group (negative mode); (**E**) G-AE group (negative mode); (**F**) Control group (negative mode).

Table S1. in vitro chemical composition characterization of G-CQDs.

NO	RT (min)	m/z	Adducts	Formula	name	Fragment
1	0.64	110.0600	$[M+H]^+$	C_6H_7NO	3-Hydroxy-2-methylpyridine	95.0362; 82.0648; 67.0414
2	0.73	131.0826	$[M-H]^-$	$C_5H_{12}N_2O_2$	L-ornithine	114.0136;107.4111;70.0295
3	0.73	175.1189	[M+H]+	$C_6H_{14}N_4O_2$	L-(+)-Arginine	158.0919;130.0971;116.0701;70.0648
4	0.78	195.0509	[M-H]-	$C_6H_{12}O_7$	Gluconic acid	160.8416;75.0084

5	0.79	387.1143	[M+FA-H]$^-$	$C_{12}H_{22}O_{11}$	Sucrose	101.0240;59.0135
6	0.80	503.1615	[M-H]$^-$	$C_{18}H_{32}O_{16}$	Manninotriose	341.1080;179.0557;59.0136
7	0.80	365.1055	[M+Na]$^+$	$C_{12}H_{22}O_{11}$	D-(+)-Maltose	275.1606;203.0521;185.0414
8	0.80	124.0073	[M-H]$^-$	$C_2H_7NO_3S$	Taurine	106.9817;79.9572
9	0.80	132.0767	[M+H]$^+$	$C_4H_9N_3O_2$	Creatine	90.0546; 72.0553; 44.0493
10	0.80	127.0389	[M+H]$^+$	$C_6H_6O_3$	5-Hydroxymethyl-2-furaldehyde	109.0283;81.0334; 69.0334; 53.0385
11	0.81	116.0705	[M+H]$^+$	$C_5H_9NO_2$	D-(+)-Proline	98.0708; 70.0648; 57.0332
12	0.83	149.0091	[M-H]$^-$	$C_4H_6O_6$	Tartaric acid	121.0278;108.0439;65.0382
13	0.83	175.0248	[M-H]$^-$, [M-H2O-H]$^-$	$C_6H_8O_6$	vitamin C	113.0237;72.9926
14	0.84	191.0560	[M-H]$^-$	$C_7H_{12}O_6$	D-(−)-Quinic acid	191.0555;87.0084; 59.0135
15	0.85	112.0505	[M+H]$^+$	$C_4H_5N_3O$	Cytosine	95.0236; 70.0648
16	0.88	136.0617	[M+H]$^+$	$C_5H_5N_5$	Adenine	119.0347;94.0396; 67.0288
17	0.88	291.1299	[M+H]$^+$	$C_{10}H_{18}N_4O_6$	Argininosuccinic acid	232.0808;175.1184;70.0648
18	0.89	217.1295	[M+H]$^+$	$C_8H_{16}N_4O_3$	Acetylarginine	158.0809;116.0703;70.0650
19	0.91	152.0566	[M+H]$^+$	$C_5H_5N_5O$	Guanine	136.0298;110.0345,
20	0.92	146.0922	[M+H]$^+$	$C_5H_{11}N_3O_2$	4-Guanidinobutyric acid	118.0647;87.0437
21	0.92	124.0392	[M+H]$^+$	$C_6H_5NO_2$	Nicotinic acid	96.0439; 80.0491
22	0.93	133.0142	[M-H]$^-$	$C_4H_6O_5$	DL-Malic acid	85.0291; 68.9953
23	0.94	115.0037	[M-H]$^-$	$C_4H_4O_4$	Maleic acid	103.0241;71.0137
24	0.95	109.0760	[M+H]$^+$	$C_6H_8N_2$	2,5-Dimethylpyrazine	81.0695; 67.0539; 53.0384
25	0.98	115.0036	[M-H]$^-$	$C_4H_4O_4$	Fumaric acid	103.2596;71.0136
26	0.99	128.0353	[M-H]$^-$	$C_5H_7NO_3$	4-Oxoproline	82.0296; 67.8163
27	0.99	124.0756	[M+H]$^+$	C_7H_9NO	2-Anisidine	96.0443; 80.0494; 42.0338
28	0.99	156.0767	[M+H]$^+$	$C_6H_9N_3O_2$	L-Histidine	138.1022;110.0709;70.0648
29	1.00	130.0499	[M+H]$^+$	$C_5H_7NO_3$	L-Pyroglutamic acid	84.0440; 70.0648
30	1.00	268.1040	[M+H]$^+$	$C_{10}H_{13}N_5O_4$	L-Adenosine	136.0612;70.0648
31	1.00	191.0197	[M-H]$^-$	$C_6H_8O_7$	Citric acid	127.0397;85.0292
32	1.02	147.0299	[M-H]$^-$	$C_9H_7O_2$	Cinnamic acid	103.0491
33	1.15	123.0440	[M+H]$^+$	$C_7H_6O_2$	Benzoic acid	105.0477;77.0386; 67.0541
34	1.22	282.0843	[M-H]$^-$	$C_{10}H_{13}N_5O_5$	Isoguanosine	150.0416;107.0363
35	1.27	284.0988	[M+H-H2O]$^+$	$C_{10}H_{13}N_5O_5$	Guanosine	152.0671;135.0305
36	1.40	117.0193	[M-H]$^-$	$C_4H_6O_4$	Succinic acid	99.9253; 73.0291
37	2.96	138.0549	[M+H]$^+$	$C_7H_7NO_2$	Trigonelline HCl	120.0438;94.0647
38	3.10	353.0877	[M-H]$^-$	$C_{16}H_{18}O_9$	1-Caffe 2-oylquinic acid	191.0552;135.0455
39	3.16	457.3674	[M+H]$^+$	$C_{30}H_{48}O_3$	Oleanolic acid	295.0415;173.5231
40	3.18	385.1138	[M-H]$^-$	$C_{18}H_{24}O_{12}$	Asperulosidic acid	89.0239; 59.0134
41	3.28	147.0439	[M+H]$^+$	$C_9H_6O_2$	Coumarin	119.0486;91.0538; 65.0382
42	3.33	153.0193	[M-H]$^-$	$C_7H_6O_4$	Protocatechuic acid	109.0294;61.1597
43	3.43	137.0244	[M-H]$^-$	$C_7H_6O_3$	4-Hydroxybenzoic acid	93.0343; 71.1718

44	3.47	483.3812	[M+Na]+	$C_{30}H_{52}O_3$	Protopanaxadiol	483.3812
45	3.51	147.0439	[M+H-H2O]+	$C_9H_8O_3$	p-Coumaric acid	147.0913;95.0488;91.0542
46	3.57	715.4273	[M+FA-H]-	$C_{36}H_{62}O_{11}$	Ginsenoside Rh6	593.3696;431.3168
47	3.68	525.1612	[M-H]-	$C_{23}H_{28}O_{11}$	Albiflorin	357.1171;121.0087
48	3.75	337.0918	[2M+H]+	$C_8H_8O_4$	Vanillic acid	173.5230;151.0383;95.0487
49	4.22	599.4052	[M+Na]+	$C_{38}H_{56}O_4$	coniferyl ferulate	599.4052
50	4.38	515.1192	[M-H]-	$C_{25}H_{24}O_{12}$	Isochlorogenic acid C	353.0867;191.0555;173.0541
51	4.70	801.4985	[M+H]+	$C_{42}H_{72}O_{14}$	Pseudoginsenoside F11	143.1061;125.0956
52	4.74	221.1899	[M+H]+	$C_{15}H_{24}O$	alpha-humulene epoxide	173.5269;95.0851
53	4.78	845.4906	[M+FA-H]-	$C_{42}H_{72}O_{14}$	Ginsenoside Rg1	637.4703;101.0237
54	4.85	187.0975	[M-H]-	$C_9H_{16}O_4$	Azelaic acid	126.0965;97.0653;57.0341
55	5.57	137.0244	[M-H]-	$C_7H_6O_3$	Salicylic acid	93.0340; 65.0393
56	6.29	285.0405	[M-H]-	$C_{15}H_{10}O_6$	3,5,7-trihydroxy-2-(4-hydroxy-phenyl)chromen-4-one	265.1265;133.0293
57	6.51	283.0612	[M-H]-	$C_{16}H_{12}O_5$	(+)-Maackiain	283.0611
58	7.68	271.0600	[M+H]+	$C_{15}H_{10}O_5$	Apigenin	173.5241;153.0176;119.0485
59	7.68	799.4851	[M-H]-	$C_{42}H_{72}O_{14}$	Ginsenoside Rf	637.4302;619.4198;101.0240
60	8.10	815.4797	[M+FA-H]-	$C_{41}H_{70}O_{13}$	20(R)-Notoginsenoside R2	769.4718;637.4303;475.3780
61	8.10	769.4744	[M-H]-	$C_{41}H_{70}O_{13}$	Ginsenoside F5	475.3780;391.2845;71.0135
62	8.12	815.4796	[M-H]-	$C_{41}H_{70}O_{13}$	Ginsenoside F3	637.4305;475.3780
63	8.21	409.3827	[M+H]+	$C_{15}H_{24}$	Bicyclogermacrene	382.0540;109.1008;95.0851
64	8.21	1108.5922	[M-H]-	$C_{54}H_{92}O_{23}$	Ginsenoside Rb1	945.5404;783.4885;221.0659;101.0239
65	8.22	325.1130	[M+H-H2O]+	$C_{12}H_{22}O_{11}$	α-Lactose	145.0489;127.0384
66	8.22	623.4517	[M+H]+	$C_{36}H_{62}O_8$	Ginsenoside CK	623.4517
67	8.22	667.4414	[M+H]+	$C_{37}H_{62}O_{10}$	Notoginsenoside T2	255.2308;173.5239;173.0855
68	8.46	829.4952	[M-H]-	$C_{42}H_{72}O_{13}$	Ginsenoside RG2	637.4305;475.3780
69	8.46	869.5248	[M+H]+	$C_{46}H_{76}O_{15}$	Koryoginsenoside R1	869.5248
70	8.47	1209.6217	[M-H]-	$C_{58}H_{98}O_{26}$	Ginsenoside Ra1	1209.6217
71	8.48	783.4901	[M-H]-	$C_{42}H_{72}O_{13}$	ginsenoside Rg3	621.4365;545.3830;101.0240
72	8.52	1077.5806	[M-H]-	$C_{53}H_{90}O_{22}$	Ginsenoside Rb3	783.4878;293.0868;101.0239
73	8.80	605.4406	[M+H]+	$C_{36}H_{60}O_7$	Ginsenoside Rh3	569.4189;533.3975;425.3770
74	8.81	1077.5799	[M-H]-	$C_{53}H_{90}O_{22}$	Ginsenoside Rb2	783.4878;293.0868;101.0239
75	9.03	813.4637	[M-H]-	$C_{41}H_{68}O_{13}$	Ginsenoside La	813.4637
76	9.16	681.4221	[M+FA-H]-	$C_{36}H_{60}O_9$	Ginsenoside Rh8	635.4169;473.3637;173.5234

77	9.48	767.4932	$[M+H]^+$	$C_{42}H_{70}O_{12}$	(20E)-Ginsenoside F4	605.4367;425.3768;189.1630
78	9.48	425.3777	$[M+H]^+$	$C_{30}H_{48}O$	Lupenone	407.3666;217.1947;98.0852
79	9.50	946.5460	$[M-H]^-$	$C_{48}H_{82}O_{18}$	Ginsenoside Re	946.5460
80	9.52	637.4322	$[M-H]^-$	$C_{36}H_{62}O_9$	Ginsenoside Rh1	161.0448;113.0238;71.0134
81	9.62	793.4377	$[M-H]^-$	$C_{42}H_{66}O_{14}$	Chikusetsu saponin IVa	631.3838;569.3838
82	11.26	683.4377	$[M+FA-H]^-$	$C_{36}H_{62}O_9$	Ginsenoside F1	683.4376
83	11.73	293.1757	$[M-H]^-$	$C_{17}H_{26}O_4$	6-Gingerol	236.1044;221.1546
84	11.94	441.3723	$[M+H]^+$	$C_{30}H_{48}O_2$	Roburic acid	109.1006;95.0855
85	11.94	817.4332	$[M+Na]^+$	$C_{42}H_{66}O_{14}$	Calenduloside F	361.0735;269.0624
86	12.02	287.2227	$[M+FA-H]^-$	$C_{15}H_{30}O_2$	methyl tetradecanoate	266.9860;173.5224
87	12.16	665.4268	$[M+FA-H]^-$	$C_{36}H_{60}O_8$	Ginsenoside Rk3	619.4182;297.2229
88	12.99	829.4952	$[M-H]^-$	$C_{42}H_{72}O_{13}$	Ginsenoside F2	621.4359;459.3831;161.0452
89	13.25	183.0804	$[M+H]^+$	$C_{13}H_{10}O$	Atractylodin	141.9202;105.0334;95.0486
90	13.90	763.4274	$[M-H]^-$	$C_{41}H_{64}O_{13}$	Momordin Ic	569.3833;71.0133
91	14.09	313.2384	$[M+FA-H]^-$	$C_{17}H_{32}O_2$	methyl (E)-hexadec-9-enoate	266.9854;202.1156
92	14.89	315.2539	$[M+FA-H]^-$	$C_{17}H_{34}O_2$	methyl hexadecanoate	266.9851;173.5284
93	15.28	200.2009	$[M+H]^+$	$C_{12}H_{25}NO$	N,N-dimethyldecanamide	102.0910;88.0754;57.0679
94	15.35	275.1652	$[M-H]^-$	$C_{17}H_{24}O_3$	Ginsenoyne C	231.1744;173.5222
95	15.41	265.1810	$[M+FA-H]^-$	$C_{15}H_{24}O$	beta-Santalenol	265.1809
96	15.60	811.4848	$[M-H]^-$	$C_{42}H_{70}O_{12}$	Ginsenoside RG4	603.4256;161.0453
97	15.60	765.4796	$[M-H]^-$	$C_{42}H_{70}O_{12}$	ginsenoside Rg5	603.4256;161.0453;101.0240
98	15.76	277.1444	$[M-H]^-$	$C_{16}H_{22}O_4$	n-Butyl phthalate	183.8339;134.0368;87.3532
99	15.82	811.4845	$[M-H]^-$	$C_{42}H_{70}O_{12}$	Ginsenoside Rg6	603.4254;161.0452
100	15.90	265.1478	$[M-H]^-$	$C_{12}H_{26}O_4S$	Dodecyl sulfate	178.9922;96.9596
101	16.36	279.2318	$[M+H]^+$	$C_{18}H_{30}O_2$	α-Eleostearic acid	109.1007;81.0695;67.0539
102	16.36	277.2172	$[M-H]^-$	$C_{18}H_{30}O_2$	a-Linolenic Acid	277.2172;121.2123
103	16.77	529.3020	$[M+FA-H]^-$	$C_{26}H_{44}O_8$	Darutoside	529.3020
104	16.82	853.4950	$[M-H]^-$	$C_{44}H_{72}O_{13}$	Ginsenoside 5	853.4949
105	16.93	665.4270	$[M+FA-H]^-$	$C_{36}H_{60}O_8$	Ginsenoside Rh4	457.3664;161.0448
106	17.93	621.4372	$[M-H]^-$	$C_{36}H_{62}O_8$	ginsenoside Rh2	90.0084
107	19.19	255.2319	$[M+H]^+$	$C_{16}H_{30}O_2$	tetradec-13-enyl acetate	173.5258;113.3501;57.0696
108	19.21	256.2634	$[M+H]^+$	$C_{16}H_{33}NO$	Hexadecanamide	173.5240;88.0752
109	19.32	313.2737	$[M+H]^+$	$C_{20}H_{34}O_2$	Linolenic acid ethyl ester	257.2471;95.0851;57.0696
110	19.52	282.2792	$[M+H]^+$	$C_{18}H_{35}NO$	Oleamide	265.2520;83.0851;69.0695

Table S2. In vivo prototype component information of G-CQDs regulating energy metabolism in mice.

NO	RT (min)	m/z	Adducts	Formula	Name	Fragment
1	0.80	127.0389	[M+H]+	$C_6H_6O_3$	5-Hydroxymethyl-2-furaldehyde	109.0283;81.0334; 69.0334; 53.0385
2	0.83	149.0091	[M-H]-	$C_4H_6O_6$	Tartaric acid	121.0278;108.0439;65.0382
3	0.84	191.0560	[M-H]-	$C_7H_{12}O_6$	D-(−)-Quinic acid	191.0555;87.0084; 59.0135
4	0.92	146.0922	[M+H]+	$C_5H_{11}N_3O_2$	4-Guanidinobutyric acid	118.0647;87.0437
5	0.95	109.0760	[M+H]+	$C_6H_8N_2$	2,5-Dimethylpyrazine	81.0695; 67.0539; 53.0384
6	1.00	191.0197	[M-H]-	$C_6H_8O_7$	Citric acid	127.0397;85.0292
7	1.02	147.0299	[M-H]-	$C_9H_7O_2$	Cinnamic acid	103.0491
8	3.45	457.3674	[M+H]+	$C_{30}H_{48}O_3$	Oleanolic acid	295.0415;173.5231
9	3.47	483.3812	[M+Na]+	$C_{30}H_{52}O_3$	Protopanaxadiol	483.3812
10	3.57	715.4273	[M+FA-H]-	$C_{36}H_{62}O_{11}$	Ginsenoside Rh6	593.3696;431.3168
11	3.75	337.0918	[2M+H]+	$C_8H_8O_4$	Vanillic acid	173.5230;151.0383;95.0487
12	4.78	845.4906	[M+FA-H]-	$C_{42}H_{72}O_{14}$	Ginsenoside Rg1	637.4703;101.0237
13	6.29	285.0405	[M-H]-	$C_{15}H_{10}O_6$	3,5,7-trihydroxy-2-(4-hydroxy-phenyl)chromen-4-one	265.1265;133.0293
14	7.68	799.4851	[M-H]-	$C_{42}H_{72}O_{14}$	Ginsenoside Rf	637.4302;619.4198;101.0240
15	8.10	769.4744	[M-H]-	$C_{41}H_{70}O_{13}$	Ginsenoside F5	475.3780;391.2845;71.0135
16	8.10	815.4797	[M+FA-H]-	$C_{41}H_{70}O_{13}$	20(R)-Notoginsenoside R2	769.4718;637.4303;475.3780
17	8.21	409.3827	[M+H]+	$C_{15}H_{24}$	Bicyclogermacrene	382.0540;109.1008;95.0851
18	8.21	1108.5922	[M-H]-	$C_{54}H_{92}O_{23}$	Ginsenoside Rb1	945.5404;783.4885;221.0659;101.0239
19	8.46	829.4952	[M-H]-	$C_{42}H_{72}O_{13}$	Ginsenoside Rg2	637.4305;475.3780
20	8.48	783.4901	[M-H]-	$C_{42}H_{72}O_{13}$	ginsenoside Rg3	621.4365;545.3830;101.0240
21	8.52	1077.5806	[M-H]-	$C_{53}H_{90}O_{22}$	Ginsenoside Rb3	783.4878;293.0868;101.0239
22	8.81	1077.5799	[M-H]-	$C_{53}H_{90}O_{22}$	Ginsenoside Rb2	783.4878;293.0868;101.0239
23	9.62	793.4377	[M-H]-	$C_{42}H_{66}O_{14}$	Chikusetsu saponin IVa	631.3838;569.3838
24	9.50	946.5460	[M-H]-	$C_{48}H_{82}O_{18}$	Ginsenoside Re	946.546
25	9.52	637.4322	[M-H]-	$C_{36}H_{62}O_9$	Ginsenoside Rh1	161.0448;113.0238;71.0134
26	11.26	683.4377	[M+FA-H]-	$C_{36}H_{62}O_9$	Ginsenoside F1	637.4299;475.3776;161.0448
27	11.72	293.1757	[M-H]-	$C_{17}H_{26}O_4$	6-Gingerol	236.1044;221.1146;205.1228
28	11.94	817.4332	[M+Na]+	$C_{42}H_{66}O_{14}$	Calenduloside F	361.0735;269.0624
29	15.35	275.1652	[M-H]-	$C_{17}H_{24}O_3$	Ginsenoyne C	231.1744;173.5222

30	15.60	765.4796	$[M-H]^-$	$C_{42}H_{70}O_{12}$	ginsenoside Rg5	603.4256;161.0453;101.0240
31	15.82	811.4845	$[M-H]^-$	$C_{42}H_{70}O_{12}$	Ginsenoside Rg6	603.4254;161.0452
32	16.36	279.2318	$[M+H]^+$	$C_{18}H_{30}O_2$	α-Eleostearic acid	109.1007;81.0695;67.0539
33	16.93	665.4270	$[M+FA-H]^-$	$C_{36}H_{60}O_8$	Ginsenoside Rh4	457.3664;61.0448
34	19.32	313.2737	$[M+H]^+$	$C_{20}H_{34}O_2$	Linolenic acid ethyl ester	257.2471;95.0851;57.0696
35	19.52	282.2792	$[M+H]^+$	$C_{18}H_{35}NO$	Oleamide	265.2520;83.0851;69.0695

Table 3. In vivo metabolism component information of G-CQDs regulating energy metabolism in mice.

NO.	RT (min)	m/z	Adducts	Formula	Drug metabolites	Transformations
M1	4.31	793.4387	$[M-H]^-$	$C_{42}H_{66}O_{14}$	Ginsenoside Rg1-H_2-H_2-H_2	Desaturation, Desaturation, Desaturation
M2	8.52	561.2918	$[M-2H]^{2-}$	$C_{54}H_{92}O_{24}$	Ginsenoside Re+O+$C_6H_{10}O_6$	Oxidation, Glucoside Conjugation
M3	8.08	650.3155	$[M-2H]^{2-}$	$C_{60}H_{102}O_{30}$	Ginsenoside Rb_1+H_2O+$C_6H_8O_6$	Hydration, Glucuronide Conjugation
M4	8.11	815.4795	$[M-H]^-$	$C_{42}H_{72}O_{15}$	Ginsenoside F_5+O+O+CH_2	Oxidation, Oxidation, Methylation
M5	9.20	343.2125	$[M-2H]^{2-}$	$C_{36}H_{64}O_{12}$	Ginsenoside Rh1+H_2O+O+O	Hydration, Oxidation, Oxidation
M6	8.66	815.4795	$[M-H]^-$	$C_{37}H_{64}O_{11}$	Ginsenoside Rh1+O+O+CH_2	Oxidation, Oxidation, Methylation
M7	18.17	640.4191	$[M-H-H_2O]^-$	$C_{38}H_{61}NO_8$	Ginsenoside Rh1-H_2O-H_2O+C_2H_3ON	Dehydration, Dehydration, Glycine Conjugation
M8	4.31	815.4795	$[M-H]^-$	$C_{42}H_{66}O_{14}$	Ginsenoside Rh4+O-H_2O+$C_6H_4O_4$	Oxidation，Dehydration, Glucuronide Conjugation
M9	19.76	501.3948	$[M-H]^-$	$C_{32}H_{54}O_4$	Protopanaxadiol+C_2H_2O	Acetylation
M10	19.58	501.3949	$[M-H]^-$	$C_{32}H_{54}O_4$	Protopanaxatriol-H_2O+H_2+C_2H_2O	Dehydration, Reduction, Acetylation
M11	19.29	501.3950	$[M-H+HAc]^-$	$C_{30}H_{50}O_2$	Protopanaxatriol-H_2O-H_2O+H_2	Dehydration, Dehydration, Reduction
M12	19.75	593.4423	$[M-H-H_2O]^-$	$C_{36}H_{60}N_4O_4$	Protopanaxatriol-H_2O-H_2+$C_6H_{12}ON_4$	Dehydration, Desaturation, Arginine Conjugation
M13	10.32	351.2177	$[M-H]^-$	$C_{20}H_{32}O_5$	Linolenic acid+O+O+C_2H_2O	Oxidation, Oxidation, Acetylation
M14	11.88	309.2071	$[M-H]^-$	$C_{18}H_{30}O_4$	Linolenic acid+O+O	Oxidation, Oxidation
M15	13.68	313.2382	$[M-H]^-$	$C_{18}H_{34}O_4$	Linolenic acid+H_2O+O+H_2	Hydration, Oxidation, Reduction
M16	15.05	586.3150	$[M-H]^-$	$C_{28}H_{49}N_3O_8S$	Linolenic acid+H_2+$C_{10}H_{17}N_3O_6S$	Reduction, GSH Conjugation
M17	16.30	311.2226	$[M-H]^-$	$C_{18}H_{32}O_4$	Linolenic acid+H_2O+O	Hydration, Oxidation
M18	16.83	305.2120	$[M-H]^-$	$C_{19}H_{30}O_3$	Linolenic acid-H_2O+O+CH_2	Desaturation, Oxidation, Methylation
M19	17.10	301.2171	$[M-H]^-$	$C_{20}H_{30}O_2$	Linolenic acid-H_2O+C_2H_2O	Dehydration, Acetylation
M20	17.15	295.2276	$[M-H]^-$	$C_{18}H_{32}O_3$	Linolenic acid+H_2O	Hydration

M21	17.68	303.2327	$[M-H]^-$	$C_{20}H_{32}O_2$	Linolenic acid-H_2O+H_2+C_2H_2O	Dehydration, Reduction, Acetylation
M22	17.72	437.2672	$[M-H]^-$	$C_{23}H_{38}N_2O_6$	Linolenic acid+O+O+$C_5H_8N_2O_2$	Oxidation, Oxidation, Glutamine Conjugation
M23	18.35	297.2433	$[M-H]^-$	$C_{18}H_{34}O_3$	Linolenic acid+H_2O+H_2	Hydration, Reduction
M24	2.58	181.0506	$[M-H]^-$	$C_9H_{10}O_4$	Cinnamic acid+H_2O+O	Hydration, Oxidation
M25	2.90	261.0074	$[M-H]^-$	$C_9H_{10}O_7S$	Cinnamic acid+H_2O+O+SO_3	Hydration, Oxidation, Sulfation
M26	5.61	179.0714	$[M-H]^-$	$C_{10}H_{12}O_3$	Cinnamic acid+H_2O+CH_2	Hydration, Methylation
M27	5.78	245.0125	$[M-H]^-$	$C_9H_{10}O_6S$	Cinnamic acid+H_2O+SO_3	Hydration, Sulfation
M28	3.32	242.9969	$[M-H]^-$	$C_9H_8O_6S$	Cinnamic acid+O+SO_3	Oxidation, Sulfation
M29	1.63	283.0682	$[M-H+HAc]^-$	$C_7H_{12}O_8$	Quinic acid+O+O	Oxidation, Oxidation
